# Supplementary material for: Patterns of late Holocene and historical extinctions on Madagascar
Source: Camb Prism Extinct. 2025 May 5;3:e9. doi: 10.1017/ext.2024.19 (PMC12179543; doi:10.1017/ext.2024.19)
Supplement: Godfrey et al. supplementary material [file S2755095824000196sup001.zip › Supplementary Table 3.pdf]

Godfrey et al. Supplementary Table 3. Inferred changes in habitat and biota with proposed climate or human triggers in the drier vegetation zones of Madagascar during the Holocene (see Figure 1 for locality details). Time of megafaunal collapse at multiple sites within the drier part of Madagascar is indicated by gray shading.

Dates[1]	Site	Nature of Change	Trigger proposed by original authors[2]	Source
Early and middle Holocene	Tsimanampesotse	Lack of stalagmite growth between the end of the Pleistocene (11,700 years ago) and ~3500 years ago suggests mean annual rainfall typically less than 300 mm/yr along the coastal southwest.	Climate	Burns et al. 2022
Beginning ~3600 years ago	Asafora	Renewed stalagmite growth suggests somewhat wetter conditions.	Climate	Faina et al. 2021
Beginning ~3500 years ago	Anaviavy Atsimo	Renewed stalagmite growth suggests somewhat wetter conditions.	Climate	Faina et al. 2024
3483 cal yr BP	Lamboharana	Early anthropogenic modification of hippopotamus femur confirmed (possible marrow extraction?).	Humans	Hixon et al. 2022 MacPhee & Burney 1991
Between ~2500 and ~1600 yr BP	Asafora	Gradual aridification, accelerating ~1700-1600 yr BP.	Climate	Faina et al. 2021
Between ~2500 and ~1500 years ago	Taolambiby	Decrease in spores of *Sporormiella* and increase in charcoal particles suggest gradual decline in faunal biomass and increase in fire.	Climate	Ramarolahy et al. 2021
Between ~2500 and 1500 yr BP	Taolambiby, Anavoha, Lamboharana, Andolonomby	Sporadic evidence of butchery or marrow extraction from bones of megaherbivores and butchery of large-bodied lemurs (e.g., *Megaladapis*).	Humans	Perez et al. 2005, Hansford et al. 2018, Rabemananjara 2020
~2050 cal yr BP	Lake Ihotry	Palm trees (especially *Bismarckia nobilis*) begin to decline in abundance.	Climate	Vallet-Coulomb et al. 2006
Beginning ~2000 years ago	Tampolove	Decline in subfossil endemic vertebrates (hippopotamuses, giant tortoises, crocodiles).	Climate	Hixon et al. 2022

~2000 years ago	Andolonomby	Abrupt increase in nitrogen isotope values for extinct lemurs suggests foraging in drier habitats.	Climate	Crowley et al. 2017
After ~2000 years ago	Tsimanampesotse	Decline in freshwater birds, likely triggered by salinization of this coastal lake.	Climate	Rasolonjatovo et al. 2021
After ~2000 years ago	Tsimanampesotse	Local extirpation of both large- and smaller-bodied vertebrates, including now-extinct lemurs.	Climate	Godfrey et al. 2021
1907.5 ±97.5 cal yr BP	Belo-sur-Mer	Decline in spores of *Sporormiella* suggests decline in large-bodied vertebrates.	Humans*	Burney et al. 2003
1700 ±135 cal yr BP	Belo-sur-Mer	Spike in charcoal microparticles suggests increase in fire.	Humans*	Burney et al. 2003
1607.5 ±92.5 cal yr BP	Andolonomby	Decline in spores of *Sporormiella* suggests decline in large-bodied vertebrates.	Humans*	Burney et al. 2003
Between ~1600 and 900 yr BP	Asafora	Sharp decline in rainfall amount with aridification peak at ~1600 yr BP followed by prolonged dry interval and second aridification peak at 1100 –1000 yr BP. Habitat shift with decline in C_3 and increase in C_4/CAM plants.	Climate	Faina et al. 2021
1574 cal yr BP	Andolonomby	Decline in *Pandanus* and other C_3 trees while sedges (Cyperaceae) increase.	Climate	Virah-Sawmy et al. 2016
1530 cal yr BP	Lake Ihotry	Diatom-inferred conductivity suggests a shift from freshwater to saline conditions.	Climate	Vallet-Coulomb et al. 2006
~1500 years ago	Andolonomby	Spike in charcoal microparticles, decline in arboreal pollen and rise in ruderal pollen.	Humans	Burney et al. 2003
By ~1500 years ago	unspecified	Genetic evidence suggests translocation from Africa to Madagascar of wild animals (bush pigs) by 1500 yr BP	Humans	Balboa et al. 2024
Beginning ~1500 years ago	Taolambiby	Vegetation shifts from dominant C_3 to dominant C_4/CAM (especially sedges).	Climate	Ramarolahy et al. 2021
Between ~1200 and 1000 cal yr BP	Andolonomby, Lamboharana, Tampolove, Tsirave	Intensification of butchery of now-extinct endemic vertebrates (e.g., *Pachylemur*, *Archaeolemur*, *Megaladapis*, *Hippopotamus*, *Mullerornis*, and *Aepyornis*)	Humans	MacPhee & Burney 1991<br>Vasey & Godfrey 2022<br>Hixon et al. 2022
By ~1100 years ago	Multiple Sites	Odds-ratio analysis confirms a dramatic megafaunal population crash. The odds of finding bones of extinct (vs. extant) species drop significantly at this time.	Climate	Faina et al. 2021

By ~1100 years ago	Antsirafaly & Soalara	Local extirpation of hippos, elephant birds, and giant arboreal lemurs (*Megaladapis*).	Climate	Godfrey et al. 2021
By ~1000 cal yr BP	Andolonomby	Virtually all trees except palms have disappeared. Palms follow soon thereafter.	Climate	Virah-Sawmy et al. 2016
Between 1000 and 900 yr BP	Asafora	Return to relatively wetter conditions.	Climate	Faina et al. 2021
~800 years ago	Tsirave	Last record of giant lemurs at lemur butchery site.	Humans	Vasey & Godfrey 2022
Between ~800 and 600 yr BP	Asafora	Dry event.	Climate	Faina et al. 2021
~600 years ago	Lake Tsizavatsy	Dry event.	Climate	Razanatsoa et al. 2021

[1]Conventional ^{14}C dates, reported in calendar years BP (cal yr BP), were calibrated or recalibrated using Calib 8.2 (Stuiver and Reimer 1993) and the Southern Hemisphere calibration curve (SHCal20; Hogg et al. 2020). Th/U dates (from stalagmites) do not require calibration and are presented in “yr BP”. Approximate dates from indirect evidence and dates from statistical analyses of large samples are provided as “years ago.”

[2]Asterisk signals that our interpretation of the trigger differs at least somewhat from that of the original authors (see main text).

Sources

Balboa RF, Bertola LD, Brüniche-Olsen A, Rasmussen MS, Liu X, Besnard G, Salmona J, Santander CG, He S, Zinner D, Pedrono M, Muwanika V, Masembe C, Schubert M, Kuja J, Quinn L, Garcia-Erill G, Stæger FF, Rakotoarivony R, Henrique M, Lin L, Wang X, Heaton MP, Smith TPL, Hanghøj K, Sinding M-H S, Atickem A, Chikhi L, Roos C, Gaubert P, Siegismund HR, Moltke I, Albrechtsen A and Heller R (2024) African bushpigs exhibit porous species boundaries and appeared in Madagascar concurrently with human arrival. *Nature Communications* **15**, 172. https://doi.org/10.1101/2023.08.23.553838

Burney DA, Robinson GS and Burney LP (2003) *Sporormiella* and the late Holocene extinctions in Madagascar. *Proceedings of the National Academy of Sciences of the USA* **100**(19), 10800-10805. https://doi.org/10.1073/pnas.1534700100

Burns SJ, McGee D, Scroxton N, Kinsley CW, Godfrey LR, Faina P and Ranivoharimanana L. (2022) Southern Hemisphere Controls on ITCZ Variability in southwest Madagascar over the past 117,000 years. *Quaternary Science Reviews* **276**, 107317. https://doi.org/10.1016/j.quascirev.2021.107317

Crowley BE, Godfrey LR, Bankoff RJ, Perry GH, Culleton BJ, Kennett DJ, Sutherland MR, Samonds KE and Burney DA (2017) Island-wide aridity did not trigger recent megafaunal extinctions in Madagascar. *Ecography: Patterns and Process in Ecology* **40**(8), 901-912. https://doi.org/10.1111/ecog.02376

Faina P, Burns SJ, Godfrey LR, Crowley BE, Scroxton N, McGee D, Sutherland MR and Ranivoharimanana L (2021). Comparing the paleoclimates of northwestern and southwestern Madagascar during the late Holocene: implications for the role of climate in megafaunal extinction. *Malagasy Nature* **15**, 108-127.

Faina P, Burns SJ, Godfrey LR, Dawson RR, McGee D, Tiger BH, Scroxton N, Ranivoharimanana L and Douglass K (2024). How wet is "wet": Comparing pluvials in southern Madagascar. *American Journal of Biological Anthropology* **183**(S77), 48.

Godfrey LR, Crowley BE, Muldoon KM, Burns SJ, Scroxton N, Klukkert ZS, Ranivoharimanana L, Alumbaugh J, Borths M, Dart R, Faina P, Goodman SM, Gutierrez IJ, Hansford JP, Hekkala ER, Kinsley CW, Lehman P, Lewis ME, McGee D, Pérez VR, Rahantaharivao NJ, Rakotoarijaona M, Rasolonjatovo HAM, Samonds KE, Turvey ST, Vasey N and Widmann P (2021). Teasing apart impacts of human activity and regional drought on Madagascar's large-bodied vertebrates: insights from new excavations at Tsimanampesotse and Antsirafaly. *Frontiers in Ecology and Evolution* **9**, 742203. https://doi.org/10.3389/fevo.2021.742203

Hansford J, Wright PC, Rasoamiaramanana A, Pérez V, Godfrey LR, Errickson D, Thompson T and Turvey ST (2018) Early Holocene human presence in Madagascar evidenced by exploitation of avian megafauna. *Science Advances* **4**, eaat6925 https://www.science.org/doi/10.1126/sciadv.aat6925

Hixon SW, Domic AI, Douglass KG, Roberts P, Eccles L, Buckley M, Ivory S, Noe S and Kennett DJ (2022) Cutmarked bone of drought-tolerant extinct megafauna deposited with traces of fire, human foraging, and introduced animals in SW Madagascar. *Scientific Reports* **12**(1), 18504. https://doi.org/10.1038/s41598-022-22980-w

Hogg AG, Heaton TJ, Hua Q., Palmer JG, Turney CS, Southon J, Bayliss A, Blackwell PG, Boswijk G, Ramsey CB and Pearson C (2020). SHCal20 Southern Hemisphere calibration, 0–55,000 years cal BP. *Radiocarbon* **62**, 759–778. https://doi.org/10.1017/RDC.2020.59

MacPhee RDE and Burney DA (1991) Dating of modified femora of extinct dwarf *Hippopotamus* from southern Madagascar: Implications for constraining human colonization and vertebrate extinction events. *Journal of Archaeological Science* 18(6), 695-706. https://doi.org/10.1016/0305-4403(91)90030-S

Perez VR, Godfrey LR, Nowak-Kemp M, Burney DA, Ratsimbazafy J and Vasey N (2005) Evidence of early butchery of giant lemurs in Madagascar. *Journal of Human Evolution* **49** (6), 722-742. https://doi.org/10.1016/j.jhevol.2005.08.004

Rabemananjara MRK (2020). Determination des effets anthropiques sur les subfossiles des Lémuriens de Sud-Ouest de Madagascar. Masters II thesis. Anthropobiologie et Developpement Durable. Université d'Antananarivo.

Ramarolahy FM, Burney DA and Godfrey LR (2021) Paleoecological evidence for late Holocene aridification from the Taolambiby subfossil site of southwestern Madagascar. *Malagasy Nature* **15**, 79-93.

Rasolonjatovo HAM, Muldoon KM, Ranivoharimanana L, Rakotoarijaona M, and Goodman SM (2021) Subfossil birds from a submerged cave in southwestern Madagascar. *Malagasy Nature* **15**, 128-140.

Razanatsoa E, Virah-Sawmy M, Woodborne S, Callanana C and Gillson L (2021) Adaptation of subsistence strategies of the southwestern Malagasy in the face of climate change. *Malagasy Nature* **15**, 41-55.

Stuiver M and Reimer PJ (1993). CALIB rev. 8. *Radiocarbon* **35**, 215-230. http://calib.org/calib/whatsnew.html

Vallet-Coulomb C, Gasse F, Robison L, Ferry L, Van Campo E and Chalié, F (2006) Hydrological modeling of tropical closed Lake Ihotry (SW Madagascar): Sensitivity analysis and implications for paleohydrological reconstructions over the past 4000 years. *Journal of Hydrology* **331**(1-2), 257-271. https://doi.org/10.1016/j.jhydrol.2006.05.026

Vasey N and Godfrey LR (2022). Lemur hunting in Madagascar's present and past: The case of *Pachylemur*. In Urbani B, Youlatos D and Antczak A (eds), *World Archeoprimatology: Interconnections of Humans and Nonhuman Primates in the Past*. Cambridge UK: Cambridge University Press, 395-416.

Virah-Sawmy M, Gillson L, Gardner CJ, Anderson A, Clark G and Haberle S (2016) A landscape vulnerability framework for identifying integrated conservation and adaptation pathways to climate change: The case of Madagascar's spiny forest. *Landscape Ecology* **31**(3), 637-654. https://doi.org/10.1007/s10980-015-0269-2
