# Supplementary material for: Patterns of late Holocene and historical extinctions on Madagascar
Source: Camb Prism Extinct. 2025 May 5;3:e9. doi: 10.1017/ext.2024.19 (PMC12179543; doi:10.1017/ext.2024.19)
Supplement: Godfrey et al. supplementary material [file S2755095824000196sup001.zip › Supplementary Figure 1.pdf]

Godfrey et al. Supplementary Figure 1.

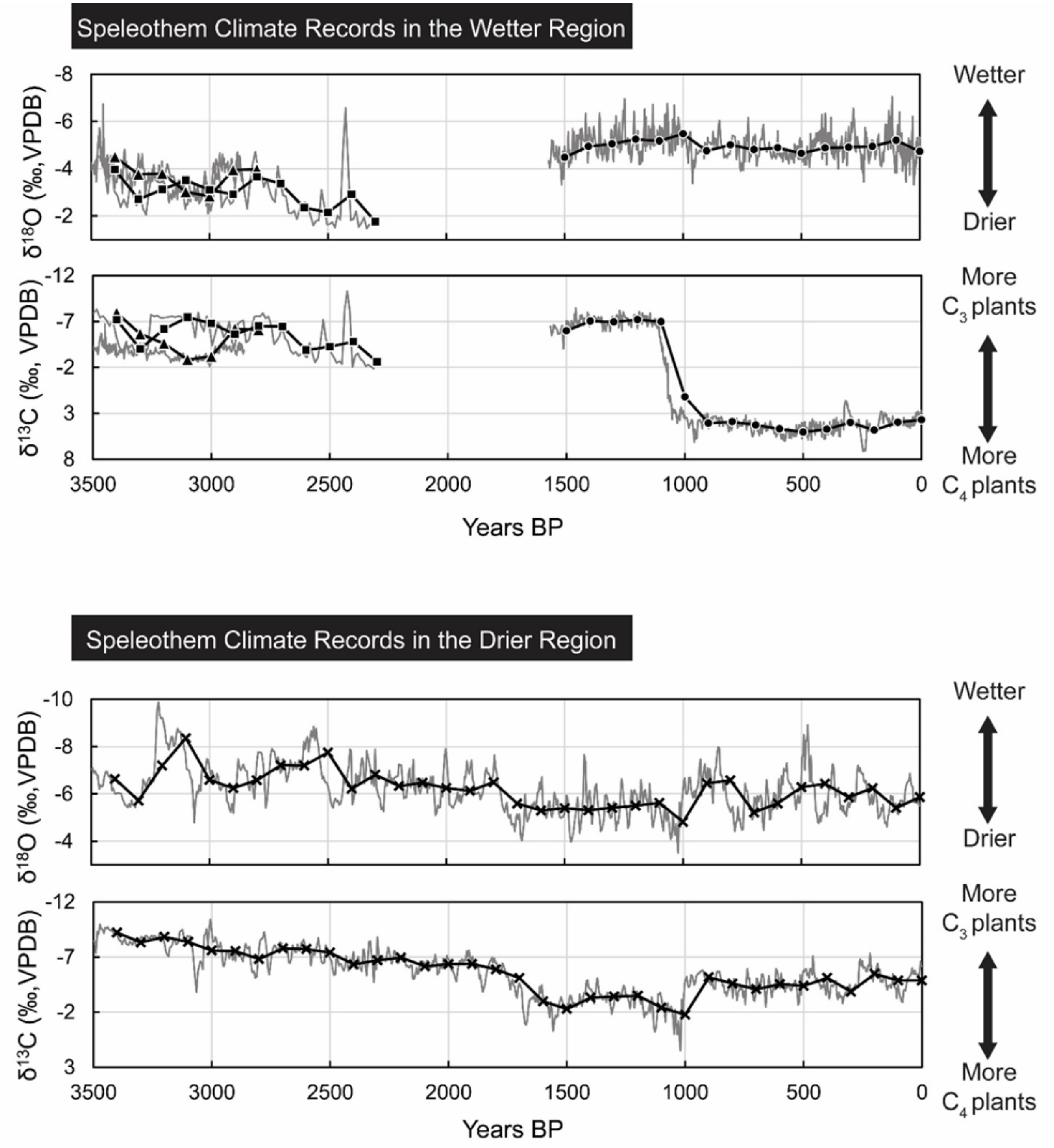

Supplementary Figure 1. High resolution speleothem records of climate from Madagascar. Climate records from the Wetter Region are based on Anjohibe stalagmites AB11, AB13, and AB2. Climate records from the Drier Region are based on Asafora stalagmite AF2. Published data are in gray and 100-year averages are in black. Wetter Region symbols are Circles (Scroxton et al., 2017); Squares (Dawson et al., 2024); and Triangles (Williams et al., 2024). Drier Region symbols are X's (Faina et al., 2021).
