# Supplementary material for: Patterns of late Holocene and historical extinctions on Madagascar
Source: Camb Prism Extinct. 2025 May 5;3:e9. doi: 10.1017/ext.2024.19 (PMC12179543; doi:10.1017/ext.2024.19)
Supplement: Godfrey et al. supplementary material [file S2755095824000196sup001.zip › Supplementary Figure 2.pdf]

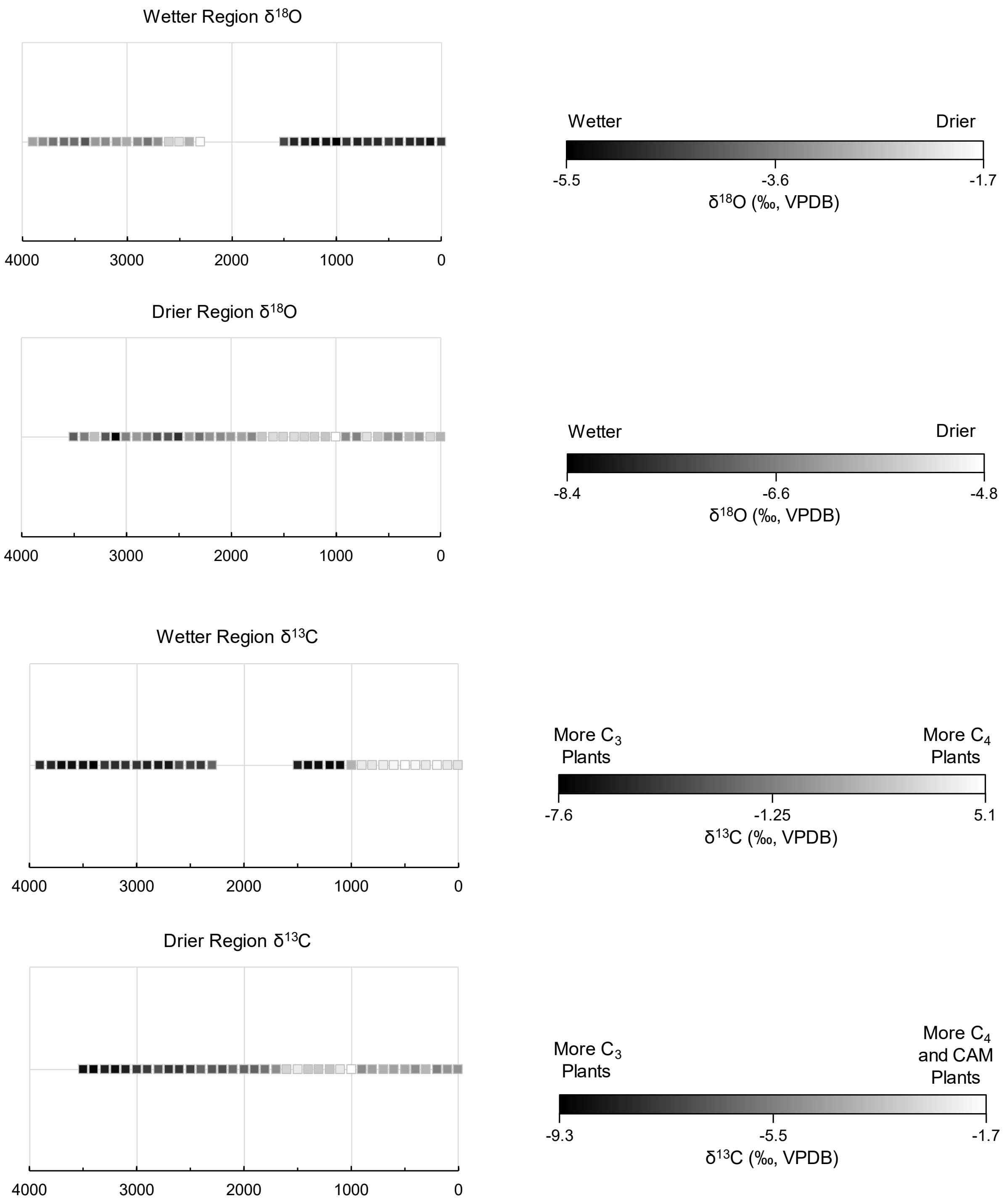

Godfrey et al. Supplementary Figure 2. One hundred-year binned average δ values of speleothem isotopic records of climate from Madagascar shown in Supplementary Figure 1

(Scroxton et al., 2017, Dawson et al., 2024, Williams et al., 2024, Faina et al., 2021). The grayscales (right) represent the $\delta^{18}O$ and $\delta^{13}C$ values and correspond to grayscales in Figure 2. The scales are different for the wetter region and the drier region due to local effects including differences in latitude (Asafora being further south) and distance from the moisture source. Clouds that have traveled further from the moisture source will likely have experienced greater rainout of the heavy isotopes, leaving the remaining moisture (and associated rainfall) with lower $\delta^{18}O$ values. Thus, even with lower rainfall amount, southern localities can have more negative $\delta^{18}O$ values than northern localities if the source of the moisture has traveled from north to south.
