# Supplementary material for: Patterns of late Holocene and historical extinctions on Madagascar
Source: Camb Prism Extinct. 2025 May 5;3:e9. doi: 10.1017/ext.2024.19 (PMC12179543; doi:10.1017/ext.2024.19)
Supplement: Godfrey et al. supplementary material [file S2755095824000196sup001.zip › Supplementary Table 1.pdf]

Godfrey et al. Supplementary Table 1. Malagasy terrestrial vertebrates that went extinct during the Holocene

Class	Order	Family	Genus/species
Mammalia	Bibymalagasia	Plesiorycteropidae	*Plesiorycteropus madagascariensis* Filhol, 1895
			Plesiorycteropus germainepetterae MacPhee, 1994
	Afrosoricida	Tenrecidae	*Microgale macpheei* Goodman, Vasey & Burney, 2007
	Chiroptera	Hipposideridae	*Hipposideros besaoka* Samonds, 2007
			Triaenops goodmani Samonds, 2007
	Carnivora	Eupleridae	*Cryptoprocta spelea* G. Grandidier, 1902
			Cryptoprocta sp. (undescribed)
	Primates	Lemuridae	*Pachylemur insignis* Filhol, 1895
			Pachylemur jullyi G. Grandidier, 1899
		Archaeolemuridae	*Archaeolemur edwardsi* Filhol, 1895
			Archaeolemur majori Filhol, 1895
			Hadropithecus stenognathus Lorenz von Liburnau, 1899
		Palaeopropithecidae	*Palaeopropithecus ingens* G. Grandidier, 1899
			Palaeopropithecus maximus Standing, 1903
			Palaeopropithecus kelyus Gommery et al. 2009
			Archaeoindris fontoynontii Standing, 1909
			Babakotia radofilai Godfrey et al. 1990
			Mesopropithecus dolichobrachion Simons et al. 1995
			Mesopropithecus globiceps Lamberton, 1936
			Mesopropithecus pithecoides Standing, 1905
		Megaladapidae	*Megaladapis edwardsi* (G. Grandidier, 1899)
			Megaladapis grandidieri Standing, 1903
			Megaladapis madagascariensis Forsyth-Major, 1894
		Daubentoniidae	*Daubentonia robusta* (Lamberton, 1934)
	Artiodactyla	Hippopotamidae	*Hippopotamus lemerlei* A. Grandidier & Milne-Edwards, 1868

			Hippopotamus laloumena Faure & Guérin, 1990
			Hippopotamus guldbergi Fauvet, Faure & Guérin, 2011
	Rodentia	Nesomyidae	*Brachytarsomys mahajambaensis* Mein *et al.* 2010
			Hypogeomys australis G. Grandidier, 1903
			Nesomys narindaensis Mein *et al.* 2010
Aves	Aepyornithiformes	Aepyornithidae	*Aepyornis hildebrandti* Burckhardt, 1893
			Aepyornis maximus I. Geoffroy St. Hilaire, 1851
			Mullerornis modestus Milne-Edwards & Grandidier, 1869
	Pelecaniformes	Phalacrocoracidae	*Phalacrocorax* sp.
	Anseriformes	Anatidae	*Alopochen sirabensis* (Andrews, 1897)
			Centrornis majori Andrews, 1897
	Falconiformes	Accipitridae	*Stephanoaetus mahery* Goodman, 1994
			Aquila sp. A
			Aquila sp. B
	Gruiformes	Mesitornithidae	*Monias* sp. (undescribed)
		Rallidae	*Hovacrex roberti* (Andrews, 1897)
	Charadriiformes	Charadriidae	*Vanellus madagascariensis* Goodman, 1996
	Cuculiformes	Cuculidae	*Coua berthae* Goodman & Ravoavy, 1993
			Coua delalandei (Temminck, 1827)
			Coua primaeva Milne-Edwards & A. Grandidier, 1895
	Coraciiformes	Brachypteraciidae	*Brachypteracias langrandi* Goodman, 2000
Reptilia	Crocodylia	Crocodylidae	*Voay robustus* A. Grandidier & Vaillant, 1872
	Testudines	Testudinidae	*Aldabrachelys abrupta* (A. Grandidier, 1868)
			Aldabrachelys grandidieri (Vaillant, 1885)
