# Supplementary material for: Patterns of late Holocene and historical extinctions on Madagascar
Source: Camb Prism Extinct. 2025 May 5;3:e9. doi: 10.1017/ext.2024.19 (PMC12179543; doi:10.1017/ext.2024.19)
Supplement: Godfrey et al. supplementary material [file S2755095824000196sup001.zip › Supplementary Table 2.pdf]

Godfrey et al. Supplementary Table 2. Inferred changes in habitat and biota with proposed climate or human triggers in the wetter vegetation zones of Madagascar during the Holocene (see Figure 1 for locality details). Time of megafaunal collapse at multiple sites within the wetter part of Madagascar is indicated by gray shading.

Dates[1]	Site	Nature of Change	Trigger proposed by original authors[2]	Source
Between 9,800 and 7,800 years ago	Anjohibe	Relatively wet period.	Climate	Voarintsoa et al. 2017
~9200 years ago	Anjohibe	Dry interval interrupts an otherwise prolonged wet period.	Climate	Dawson et al. 2024
Between 9,000 and 8,000 years ago	Anjohibe	Two relatively wet periods (~8,300 and 8,200 years ago) attributed to meltwater pulse in North Atlantic and the global "8.2 ka" event	Climate	Voarintsoa et al. 2019
Between 8,500 and 7,500 years ago	Anjohibe	Agreement with previously reported relatively wet 8.2 ka event but with 2-stage structure observed at ~8,200 and ~8,100 years ago	Climate	Duan et al., 2021
Between 7,800 and 1,600 years ago	Anjohibe	Lack of stalagmite growth suggests dry mid-Holocene climate.	Climate	Voarintsoa et al. 2017
Between ~6,000 and. 4,000 years ago	Anjohibe	Shift from early Holocene wet conditions to drier conditions with transition between ~6000 and 4000 years ago.	Climate	Dawson et al. 2024
~5500 years ago	Lake Maudit	Shift from wetter to drier conditions.	Climate	Teixeira et al. 2021
4800—4600 yr BP	Anjohibe	Dry period.	Climate	Wang et al. 2019
Between 4310 and 3930 yr BP	Anjohibe/ Anjohikely	Two pronounced dry intervals.	Climate	Scroxton et al. 2023
Between 4300 and 4000 yr BP	Anjohibe	Dry interval.	Climate	Wang et al. 2019
~3200 to 3000 yr BP	Anjohibe	Dry interval.	Climate	Williams et al. 2024

Between ~2500 and 1500 years ago	Anjohibe	Minor landscape change believed by authors to have been human-induced.	Humans*	Wang et al. 2019
Between ~2500 and 2000 years ago	Anjohibe	Megaherbivores (hippopotamuses and elephant birds) disappear locally.	Unclear*	Hansford et al. 2021
Common Era (2000 BP to present)	Anjohibe	Comparison of Common Era (CE) rainfall to Last Glacial Maximum reveals rainfall on average lower during the CE.	Climate	Tiger et al. 2023
Starting ~2000 cal yr BP	Lake Alaotra	Habitat transformation with decrease in trees and increase in upland herbs and grasses	Humans*	Broothaerts et al. 2023
1607.5 ± 137.5 cal yr BP	Lake Komango	Spike in charcoal microparticles suggests an increase in fire.	Humans*	Burney et al. 2004
Starting ~1600 years ago	Anjohibe	Return of relatively wet conditions.	Climate	Voarintsoa et al. 2017
Starting ~1600 years ago	Anjohibe	Return of relatively wet conditions.	Climate	Scroxton et al. 2017
~1300 cal yr BP	Lake Amparihibe	Abrupt environmental change from rain forest to C_4 grass-dominated, fire-disturbed landscape	Humans	Reinhardt et al. 2022
1237.5 ± 167.5 cal yr BP	Lake Kavitaha	Spike in charcoal microparticles suggests an increase in fire.	Humans	Burney et al. 2003
Beginning ~1200 years ago	Anjohibe	Vegetation increasingly dominated by savanna, despite no significant change in rainfall.	Humans	Railsback et al. 2020
1125 ± 170 cal yr BP	Lake Tritrivakely	Spike in charcoal microparticles suggests an increase in fire.	Humans	Burney et al. 2003
1100—1000 yr BP	Anjohibe	Abrupt shift from C_3 plant-dominated to C_4 plant-dominated landscape, despite no significant change in rainfall.	Humans	Burns et al. 2016
1100 —1000 yr BP	Anjohibe	Intensive sampling of stalagmite proxies for rainfall ($\delta^{18}O$) and ground vegetation ($\delta^{13}C$) confirms anthropogenic habitat transformation.	Humans	Scroxton et al. 2017
~1000 years ago	Multiple sites	Odds-ratio analysis reveals dramatic megafaunal population loss. The odds of finding bones of extinct (vs. extant) species drop significantly at this time.	Humans	Faina et al. 2021
1000 ± 95 cal yr BP	Lake Amparihibe	Spike in microscopic charcoal particles, lake eutrophication and spike in spores of *Sporormiella* suggest fire, pastoralism.	Humans	Burney, 1999<br>Burney et al. 2003

After ~1000 cal yr BP	Lake Amparihibe	Forest/grassland mosaic remains impacted by frequent fires.	Humans	Reinhardt et al. 2022
1000 – 700 cal yr BP	Lake Tritrivakely	Shift from wooded savanna to grass-dominated savanna.	Humans	Gasse & Van Campo 1998
~900 cal yr BP	Lake Maudit	Precipitous decline in pollen of evergreen forest plant taxa and simultaneous sharp rise in charcoal microparticles.	Humans	Teixeira et al. 2021
Starting ~1000 years ago	Anjohibe	Wholesale change in carbon isotopes of large-bodied vertebrates reveals consumption of C_4 plants by introduced species.	Humans	Crowley & Samonds 2013
815 ± 145 cal yr BP	Lake Kavitaha	Increase in spores of *Sporormiella*, likely reflecting the introduction of cattle.	Humans	Burney et al. 2003
~790 cal yr BP	Lake Mitsinjo	Shift from wooded savanna to grass-dominated savanna.	Humans	Matsumoto & Burney, 1994
645 ± 150 cal yr BP	Benavony	Spike in microscopic charcoal particles, indicating fire.	Humans	Burney et al. 2003
~500 cal yr BP	Lake Mitsinjo	Increase in ruderal pollen.	Humans	Matsumoto & Burney, 1994

[1]Conventional ^{14}C dates, reported in calendar years BP (cal yr BP), were calibrated or recalibrated using Calib 8.2 (Stuiver and Reimer 1993) and the Southern Hemisphere calibration curve (SHCal20; Hogg et al. 2020). Th/U dates (from stalagmites) do not require calibration and are presented in "yr BP". Approximate dates from indirect evidence or from multiple data points combined are provided as "years ago."

[2]Asterisk signals that our interpretation of the trigger differs at least somewhat from that of the original authors (see main text).

Sources

Broothaerts N, Razanamahandry VF, Brosens L, Campforts B, Jacobs L, Razafimbelo T, Rafolisy T, Verstraeten G, Bouillon S and Govers G (2023) Vegetations changes and sediment dynamics in the Lake Alaotra region, central Madagascar. *The Holocene* **33**(4), 459-470. https://doi.org/10.1177/0959686221145376

Burney DA (1999) Rates, patterns, and processes of landscape transformation and extinction in Madagascar. In MacPhee RDE (ed), *Extinction in Near Time*. New York: Kluwer Academic/ Plenum Press, 145-164.

Burney DA, Burney LP, Godfrey LR, Jungers WL, Goodman SM, Wright HT and Jull AJT (2004) A chronology for late prehistoric Madagascar. *Journal of Human Evolution* **47**(1-2), 25-63. https://doi.org/10.1016/j.jhevol.2004.05.005

Burney DA, Robinson GS and Burney LP (2003) *Sporormiella* and the late Holocene extinctions in Madagascar. *Proceedings of the National Academy of Sciences of the USA* **100**(19), 10800-10805. https://doi.org/10.1073/pnas.1534700100

Burns SJ, Godfrey LR, Faina P, McGee D, Hardt B, Ranivoharimanana L and Randrianasy J (2016) Rapid human-induced landscape transformation in Madagascar at the end of the first millennium of the Common Era. *Quaternary Science Reviews* **134**, 92-99. https://doi.org/10.1016/j.quascirev.2016.01.007

Crowley BE and Samonds KE (2013) Stable carbon isotope values confirm a recent increase in grasslands in northwestern Madagascar. *The Holocene* **23**(7), 1066-1073. https://doi.org/10.1177/0959683613484675

Dawson RR, Burns SJ, Tiger BH, McGee D, Faina P, Scroxton N, Godfrey LR and Ranivoharimanana L. (2024) Zonal control on Holocene precipitation in northwestern Madagascar based on a stalagmite from Anjohibe. *Scientific Reports* **14**, 5496. https://doi.org/10.1038/s41598-024-55909-6

Duan P, Li H, Sinha A, Voarintsoa NRG, Kathayat G, Hu P, Zhang H, Ning Y and Cheng H (2021) The timing and structure of the 8.2 ka event revealed through high-resolution speleothem records from northwestern Madagascar. *Quaternary Science Reviews* **268**, 107104. https://doi.org/10.1016/j.quascirev.2021.107104

Faina P, Burns SJ, Godfrey LR, Crowley BE, Scroxton N, McGee D, Sutherland MR and Ranivoharimanana L (2021). Comparing the paleoclimates of northwestern and southwestern Madagascar during the late Holocene: implications for the role of climate in megafaunal extinction. *Malagasy Nature* **15**, 108-127.

Gasse F and Van Campo E (1998) A 40,000-yr pollen and diatom record from Lake Tritrivakely, Madagascar, in the southern tropics. *Quaternary Research* **49**(3), 299-311. https://doi.org/10.1006/qres.1998.1967

Hansford J, Lister AM, Weston EM and Turvey ST (2021) Simultaneous extinction of Madagascar's megaherbivores correlates with late Holocene human-caused landscape transformation. *Quaternary Science Reviews* **263**, 106996 https://doi.org/10.1016/j.quascirev.2021.106996

Hogg AG, Heaton TJ, Hua Q., Palmer JG, Turney CS, Southon J, Bayliss A, Blackwell PG, Boswijk G, Ramsey CB and Pearson C (2020). SHCal20 Southern Hemisphere calibration, 0–55,000 years cal BP. *Radiocarbon* **62**, 759–778. https://doi.org/10.1017/RDC.2020.59

Matsumoto K and Burney DA (1994) Late Holocene environments at Lake Mitsinjo, northwestern Madagascar. *The Holocene* **4**(1), 16-24. https://doi.org/10.1177/095968369400400103

Railsback LB, Dupont LA, Liang F, Brook GA, Burney DA, Cheng H and Edwards RL (2020) Relationships between climate change, human environmental impact, and megafaunal extinction inferred from a 4000-year multi-proxy record from a stalagmite from northwestern Madagascar. *Quaternary Sciences Reviews* **234**, 106244. http://dx.doi.org/10.1016/j.quascirev.2020.106244

Reinhardt AL, Kasper R, Lochner M, Bliedtner M, Krahn KJ, Haberzettl T, Shumilovskikh L, Rahobisoa J-J, Zech R, Favier C, Behling H, Bremond L, Daut G and Montade V (2022) Rain forest fragmentation and environmental dynamics on Nosy Be Island (NW Madagascar) at 1300 cal BP is attributable to intensified human impact. *Frontiers in Ecology and Evolution* **9**, 783770. https://doi.org/10.3389/fevo.2021.783770.

Scroxton N, Burns SJ, McGee D, Hardt B, Godfrey LR, Ranivoharimanana L and Faina P (2017) Hemispherically in-phase precipitation variability over the last 1700 years in a Madagascar speleothem record. *Quaternary Science Reviews* **164**, 25-36. http://dx.doi.org/10.1016/j.quascirev.2017.03.017

Scroxton N, Burns SJ, McGee D, Godfrey LR, Ranivoharimanana L, Faina P and Tiger BH (2023) Hydroclimate variability in the Madagascar and Southeast African summer monsoons at the Mid- to Late-Holocene transition. *Quaternary Science Reviews* **300**, 107874. https://doi.org/10.1016/j.quascirev.2022.107874

Stuiver M and Reimer PJ (1993). CALIB rev. 8. *Radiocarbon* **35**, 215-230. http://calib.org/calib/whatsnew.html

Teixeira H, Montade V, Salmona J, Metzger J, Bremond L, Kasper T, Daut G, Rouland S, Ranarilalatiana S, Rakotondravony R, Chikhi L, Behling H and Radespiel U (2021) Past environmental changes affected lemur population dynamics prior to human impact in Madagascar. *Communications Biology* **4**(1), 1084. https://www.nature.com/articles/s42003-021-02620-1

Tiger BH, Burns SJ, Dawson RR, Scroxton N, Godfrey LR, Ranivoharimanana L, Faina P and McGee DA (2023) Zonal Indian Ocean Variability Drives Millennial-Scale Precipitation Changes in Northern Madagascar. *Paleoceanography and Paleoclimatology* 38 (11), e2023PA004626
https://doi.org/10.1029/2023PA004626

Voarintsoa NRG, Railsback LB, Brook GA, Wang L, Kathayat G, Cheng H, Li XL, Edwards RL, Rakotondrazafy AFM and Razanatseheno MOM (2017) Three distinct Holocene intervals of stalagmite deposition and nondeposition revealed in NW Madagascar, and their paleoclimate implications. *Climate of the Past* **13**(12), 1771-1790. https://doi.org/10.5194/cp-13-1771-2017

Voarintsoa, NRG, Matero ISO, Railsback LB, Gregoire LJ, Tindall J, Sime L, Cheng H, Edwards RL, Brook GA, Kathayat G, Li X, Rakotondrazafy AFM and Razanatseheno MOM (2019) Investigating the 8.2 ka event in northwestern Madagascar: insight from data-model comparisons *Quaternary Science Reviews* **204**, 172-186. https://doi.org/10.1016/j.quascirev.2018.11.030

Wang L, Brook GA, Burney DA, Voarintsoa NRG, Liang F, Cheng H and Edwards RL (2019) The African Humid Period, rapid climate change events, the timing of human colonization, and megafaunal extinctions in Madagascar during the Holocene: Evidence from a 2m Anjohibe Cave stalagmite. *Quaternary Science Reviews* **210**, 136-153. https://doi.org/10.1016/j.quascirev.2019.02.004

Williams BL, Burns SJ, Scroxton N, Godfrey LR, Tiger BH, Yellen B, Dawson RR, Faina P, McGee D and Ranivoharimanana L (2024) A speleothem record of hydroclimate variability in northwestern Madagascar during the mid-late Holocene. *The Holocene* **34**(5), 593-603. https://doi.org/10.1177/09596836231225725
